# Bridging Emotion in the Classroom with Child Understanding: Initial Findings from Linguistic Analysis for SEL Programs

**DOI:** 10.3390/bs16091482

**Published:** 2026-08-25

**Authors:** Aneyn M. O’Grady, Sonali Nag

**Affiliations:** Department of Education, University of Oxford, Oxford OX2 6PY, UK; sonali.nag@education.ox.ac.uk

**Keywords:** social–emotional learning, emotion understanding, thematic analysis, middle childhood, emotion definitions

## Abstract

Research on how children develop emotion concepts is vital to successful, culturally sensitive social–emotional learning (SEL) programs that account for individual differences in the development of SEL skills. This study presents a thematic analysis of child emotion definitions and potential implications for SEL programs as an exploration of how children are taught about emotions and to center child voice in SEL research. It is the first, to the researchers’ knowledge, to link child emotion representation with SEL programs by analyzing emotion-focused program content through the lens of a child’s emotional representational system. A total of 1239 definitions produced by 49 children ages 8 to 11 (*M_age_* = 9.24; *SD* = 0.75; 46.9% Female; 69% multilingual) were analyzed. Eight themes were identified in the definition corpus via thematic analysis and used as a framework to which 38 SEL programs were mapped. A majority of SEL programs addressed the following themes in the child-informed emotion framework: ‘interpersonal dynamics’ (addressed by 97% of programs), ‘cognitive states’ (95%), ‘performing an activity’ (87%), and ‘observable signs’ (71%) that referred to physical/sensory experience. When defining emotions, over 80% of children mentioned ‘interpersonal dynamics’ (e.g., rejection), ‘experiencing a non-social situation’ (e.g., a disappointing event), and ‘observable signs’ (e.g., sleeping). Findings offer preliminary insight into how a sample of children in middle childhood describe and make meaning of emotions, and prompt an exploratory discussion on emotion-focused content in SEL programs for middle childhood. Definitions collected constitute an emotion definition database available for future research on child emotion language.

## 1. Introduction

Over the past 20 years, social–emotional learning (SEL) has taken root in schools to promote academic performance and student well-being. This has been reinforced by global calls to reorient education policy and practice toward developing healthy psychosocial environments that improve learning and support students to thrive, as well as to achieve sustainable development goals tied to providing a quality education for all.

Social–emotional development comprises skills that can be trained as supported by evidence from school-based, researcher-developed programs to promote social–emotional skills, also referred to as training studies (Gavazzi & Ornaghi, 2011; Hofmann et al., 2016; Pons et al., 2019; Richard et al., 2021; Sprung et al., 2015), and from meta-analyses reporting a positive impact of SEL programs on social–emotional competence in addition to higher academic performance (Blewitt et al., 2018; Corcoran et al., 2018; Durlak et al., 2011; Ha et al., 2025; Hung et al., 2026; Shi et al., 2025; Shi & Cheung, 2024). Still, much of the evidence is derived using measures that assess social–emotional skills as a composite (Mckown, 2017). Questions of ‘how’ to explain outcomes by linking them to program components, and ‘for whom’ SEL programs are effective remain (Durlak et al., 2022).

Reviews of ‘effective’ programs are useful to guide practitioners in the selection of SEL programs, but additional lines of research remain underdeveloped at present to identify and describe causal mechanisms that explain program impact on children and youth. In turn, linking specific SEL program components to improved student outcomes requires deeper understanding of social–emotional development trajectories across school-age years. We report on a novel approach to analyzing SEL programs for a specific age band at the program component level through the lens of children’s emotion understanding and language.

Examining SEL program content from a developmental perspective can answer questions of ‘how’ and ‘for whom’ to help ensure SEL programs are age-appropriate, evidence-based, and relevant to the context and communities involved. As Denham (2018) advances, adopting a developmental lens to track how skills progress in children and youth allows us to identify both “what changes” and “what stays the same” as children grow, moving away from a “one-size-fits-all” approach that can render assessments, standards, and frameworks out of sync with the reality of how a child develops.

Research on causal mechanisms in SEL from a developmental perspective can further address the calls for SEL programs to be culturally relevant (Jacob, 2021; see Lim et al., 2024 and McCallops et al., 2019 for meta-analysis and systematic review), connected to the authentic experiences of learners (Hayashi et al., 2022) and used in service of creating equitable learning conditions (Jagers et al., 2019; Mahoney et al., 2021). These calls are echoed in the updated definition of SEL put forth by the Collaborative for Academic, Social, and Emotional Learning (CASEL, 2020) that underscores the need for SEL research and practice to account for the impact of environment on social–emotional development and learning, including relationships and individual, lived realities. Furthermore, access to data on children’s experience of emotions and child-informed frameworks for social–emotional development can both enrich understanding of how SEL programs impact students’ development and potentially encourage alignment, as needed, between SEL lesson content and students’ experience to better support program engagement and relevance.

### 1.1. What Is Emotion Understanding and Its Link with Language?

Emotion understanding refers to the ability to identify, interpret, and communicate about emotions (whether of the self and/or others) and, as such, includes knowledge of emotion language or emotion vocabulary—the words learned to express emotional states (Castro et al., 2016; Denham, 1986, 1998; Harris, 1989; Lewis, 2016; Parrigon et al., 2015). Harris (2008) adds that emotion understanding relates to the way a child may predict or explain emotions that they and others experience. An individual’s emotion understanding is inherently linked to their concepts of emotion—abstract representations that include knowledge of the nature of emotions, their potential causes/external triggers, linked physiological reactions, subjective feelings, potential action impulses resulting from an emotional state, and awareness of strategies to regulate emotions (Manstead, 1994; Pons et al., 2004; Saarni et al., 1998).

There are differing views on how to define an emotion, but here we understand an ‘emotion’ to have multiple components as advanced by Scherer (2005) including an appraisal (the cognitive component), bodily symptoms (the neurophysiological component), action tendencies (the motivational component), a facial and vocal expression (the motor expression component), and an emotional experience (the subjective feeling component). As such, ‘feeling’ is used to refer to the subjective experience of a specific ‘emotion’ and does not constitute the conceptual representation of the emotion itself (the emotion concept). This distinction is of particular concern to this study in its focus on which words children use to describe emotions and their emotional experiences—internal states that have a conceptual representation that is non-verbalized but nevertheless linked to specific emotion words.

Child development research on emotion understanding can be especially beneficial to inform SEL program content, as SEL programs are often designed to promote emotional awareness—both within the self and of others—as part of a broader aim to promote social skills and positive social integration. This design commonality across SEL programs underscores the importance of identifying emotions and labeling internal experiences as a foundation for social interaction and cognition.

A significant body of research evidence with children reports a positive association between language and emotion understanding (de Rosnay & Harris, 2002; Ornaghi & Grazzani, 2013; Ruffman et al., 2003; Tsou et al., 2023) as well as with social skills (Cutting & Dunn, 1999; Grazzani et al., 2018; Sarmento-Henrique et al., 2020). Knowledge of emotion vocabulary (emotion word learning) has been found to be an important anchor for organizing emotion concepts (as part of concept learning), especially for young children (e.g., Ogren & Sandhofer, 2022).

Level of emotion vocabulary has also been found to account for individual differences in emotion understanding (which includes conceptual representation) for children ages 4 to 11, specifically in their abilities to recognize and regulate emotions (Streubel et al., 2020). Still, a child’s acquisition of an emotion word does not necessarily mean they are able to use and understand the emotion word in an adult sense (Ameel et al., 2008; Saji et al., 2011). Furthermore, Nook et al. (2017) found that it was general language skill that mediated the development of more complex emotion concepts from child to adult understandings, and not the level of emotion vocabulary.

Disentangling concept learning from word learning across development is difficult (Gopnik & Meltzoff, 1997), but more research is needed on the development of emotion concepts across age bands to understand differences between different groups and to clarify the influence of additional factors such as language. Greater understanding of how children think about emotions at different ages can help to structure both the theoretical underpinnings of how emotional learning is conceptualized in SEL and emotion-focused SEL program content so that it is aligned with developmental expectations.

### 1.2. Analyzing Child Emotion Language

Previous research has analyzed child-produced emotion language, whether verbally or in written form, to identify internal representations of emotions. For example, Nook et al. (2020) assessed emotion vocabulary knowledge through a definitions task, with responses coded for levels of comprehension and abstraction. Within the realm of mental health, research has focused on language patterns as an indicator of well-being, analyzing natural emotion vocabularies to understand individual descriptions of traumatic events (Margola et al., 2010) to uncover how individuals are processing emotions.

Emotion language studies often adopt the use of diaries as a data source, and diaries have also been incorporated into SEL programs. For example, Mortari et al. (2024) conducted a qualitative study of “The *Nous* project”—an SEL program designed to promote emotional self-understanding that required children ages 9 to 10 (Grade 4) to keep a diary of their emotional lives in the form of a narration of emotions experienced throughout a day. Diary entries were then analyzed by the children, again in writing, through reflective exercises.

However, the purpose of the present research was to describe how a group of children understand emotions from a more generalized perspective—that is to say, at the level of definitions as opposed to capturing their ability to self-reflect on their lived emotional experiences. Studying emotion vocabulary alone (whether in terms of vocabulary size or emotion word usage) is insufficient to capture a child’s full understanding of an emotion that is potentially richer as children grow and gain more experiences. Children completing open-ended tasks where they can verbally describe emotional states, as was performed in the present study, can offer insight into how children spontaneously think about emotions. To the researchers’ knowledge, there is no existing database of child emotion definitions transcribed verbatim that could provide insights into how children think about and understand emotions at different ages.

### 1.3. The Importance of Middle Childhood

Middle childhood is a dynamic period both developmentally and academically for education research (Manning, 1998) that can be meaningfully supported by SEL program participation. From a developmental perspective, children ages 8 to 11 continue to acquire important social–emotional skills, including both advanced theory of mind (Peterson & Wellman, 2019; Wellman et al., 2011) and emotion concept differentiation (Eccles, 1999), in addition to strategic emotion regulation (Eisenberg & Morris, 2002) and a burgeoning moral sense (Christner et al., 2020; Kingsford et al., 2022). A reduction in egocentric errors on communication tasks is also observed in middle childhood that continues throughout adolescence—indicative of the child’s increasing ability to take the perspective of others (Wang et al., 2016), which is an important building block for social-problem solving, understanding the emotions of others, and empathy (whether cognitive or affective).

Within the Pons model of emotion understanding (Pons et al., 2004), derived from research literature on child emotion regulation and emotion understanding, children in middle childhood (ages 8 to 11) are understood to have acquired the following: the abilities to identify and label emotions; an understanding that emotions can be caused by external events; an understanding that the intensity of emotional experience can diminish over time but aspects of a present situation can give rise to emotional states experienced in the past (e.g., seeing a picture of a dead relative); an understanding that different individuals can have different emotional reactions to the same stimulus depending on their individual desires and beliefs about a situation; and an understanding that people’s outward, that is to say ‘externalized,’ display of emotion does not necessarily match their internal states (e.g., understanding people can smile and behave positively although they feel sad).

In addition, the Pons model considers children ages 8 to 11 to be in the third and final main developmental phase of emotion understanding, the ‘reflexive’ phase, where they have developed an understanding that emotional states can be regulated with different strategies, and are beginning to develop an understanding that one can experience emotions simultaneously, even those that are contradictory, as well as that emotions are linked to morality—specifically, that actions or behaviors considered morally reprehensible give rise to negative emotional states, and that those deemed morally laudable lead to positive emotional states.

Middle childhood is also an important developmental period for language skills, as it is accompanied by the development of more complex syntactic (Frizelle et al., 2018) and grammatical abilities (Muter & Snowling, 1997), and deeper semantic understanding (Deckert et al., 2019). Semantic understanding refers to the ability to comprehend the meaning of words, sentences, and other forms of communication, including understanding relationships between concepts and the context in which language is used (how words relate to each other), and being able to make inferences about intended meaning that goes beyond the ability to recognize words themselves.

Regarding the development of emotion language, children ages 8 to 11 have a decent level of emotion vocabulary to describe their emotional experiences compared to younger ages. Baron-Cohen et al. (2010) found the emotional lexicon in English doubled every two years between the ages of 4 and 11 and then leveled off from ages 12 to 16. However, the use of emotion words within middle childhood is still not the same as that of adults. Grosse et al. (2021) did find a convergence between how adults and children ages 10 and 11 used emotion words, with older children producing more emotion words than younger children, but it was still to a lesser degree than adults. The underlying conceptual structure that links understanding of emotions to emotion words can also continue to adapt with age even without a consistent, progressive increase in emotion vocabulary. For example, a plateau in emotion vocabulary was also found by Nook et al. (2020) at age 11 in their study of participants ages 4 to 25, but emotion word knowledge was found to increase with age.

From an educational perspective, children in middle childhood are in their final years of primary school—a prime opportunity to develop social–emotional skills that can be drawn upon throughout adolescence to allow for a positive transition to secondary school and beyond (Klein & Englund, 2021; Okano et al., 2019). Panayiotou et al. (2019) found a child’s level of social–emotional competence in Grade 3 predicted levels of social connectedness and mental health difficulties in Grade 4, with mental health difficulties in Grade 4 predicting and mediating academic achievement in Grade 5 (controlling for gender and prior academic performance).

Furthermore, longitudinal evidence suggests that social–emotional skills developed in childhood impact later outcomes, such as postsecondary performance and adjustment, as well as adult levels of depression (Domitrovich et al., 2017). Declines in emotional well-being (Anderson et al., 2000; Barber & Olsen, 2004), academic achievement, and achievement goals (Paulick et al., 2013) have also been reported upon secondary school entry (see Evans et al., 2018 for a review of the academic and psychological impact of the transition to secondary school), that is to say, the period following middle childhood. An accurate description of how children understand and communicate emotions in middle childhood can potentially inform SEL programs implemented in the final years of primary school to unpack their potential to both mitigate negative transitions to secondary school and lay the foundation for social–emotional skills that students can draw upon throughout adolescence and beyond.

### 1.4. The Present Study

This study explored how a sample of children ages 8 to 11 talk about emotions to glean how they think about emotions, using preliminary findings as a frame to discuss emotion-focused content in SEL programs for middle childhood. Child-produced emotion definitions and SEL program content were analyzed to address two research questions: Are there common topics children reference when defining emotions in middle childhood? Do SEL programs for the same age band (i.e., middle childhood) also address these topics in their emotion-focused content?

Child emotion definitions provide a meaningful window into children’s conceptual representation of emotions, spontaneously shared and with minimal influence from researchers (besides their selecting which emotions are to be defined by children). The act of providing a definition requires the child to select which information they find most useful or effective to describe a given emotion. As such, thematic analysis of child emotion definitions at a semantic level provides a starting point to not only describe how children understand, experience, and think about emotions, but also to identify which information might be central or most salient to actual children when it comes to emotions. This differs from children’s reflections and reactions to emotions in pre-defined narratives and tasks that necessarily present a particular view or understanding of emotions that can shape and even bias how children respond in turn.

Findings from the study’s first research question were used to create an emotion framework to which SEL programs identified by a previous systematic review (O’Grady & Nag, 2024) were then mapped. The mapping exercise was used to address the second research question. The goal of comparing emotion-focused SEL program content with a child-informed emotion framework is to set a precedent for bridging the design of SEL program content with child emotion understanding and experience from a developmental perspective. Findings can prompt an initial reflection on emotion-focused content in SEL programs. Using child emotion definitions as the basis for the study’s emotion understanding framework was part of achieving the study’s additional aim to center child voice in SEL research.

Evidence speaking to differences depending on age band and how emotional skill relates to social skill across development is a vital foundation for research concerned with the causal impact of SEL programs. Although this study cannot speak to the causal impact of SEL programs, the study findings aim to complement existing systematic reviews of SEL programs that often consider the impact of a program as a whole and not at the level of individual program components; do not consistently differentiate impact by age band or separate the impact on the development of social skills from that on emotional skills; and often lack the perspective of students participating in SEL programs.

This study is the first to the researchers’ knowledge to (1) provide a database of child-produced emotion definitions and a description of common topics to relay how children think about emotions in middle childhood; and (2) link child emotion representation with emotion-focused content found in SEL programs. This approach can advance existing SEL program evaluation by encouraging analysis to focus on the component level of programs (meaning lesson plans, aims, and activities) for a constrained, developmentally pre-defined age band, which is beneficial to future research concerned with explaining SEL program impact. This study’s analytic approach can also add a layer of framing for SEL program design and impact that incorporates students’ lived experiences through their emotion definitions.

## 2. Materials and Methods

### 2.1. Participants

Participants were 49 typically developing children enrolled in primary school grades 3 to 5 (*M_age_* = 9.24; *SD* = 0.75; 46.9% Female). Roughly 31% of the sample was monolingual (speaking English) (*n* = 15), and 69% were multilingual (*n* = 34), with the following languages spoken at home (in addition to English): Arabic, Chinese, Dutch, French, German, Irish, Italian, Japanese, Kinyarwanda, Polish, Portuguese, Russian, Spanish, Ugandan, and Welsh. Nine participants were only children, with the remainder of the sample (*n* = 40) having at least one sibling. Demographic data on the language background and number of siblings of participants were collected as part of a different study (to be reported on in a separate research article) and not used as part of the thematic analysis reported on here. Information on socio-economic status (SES) of participants was not collected as this was an exploratory qualitative study concerned with discovering potential trends in the focus of emotion definitions produced by children ages 8 to 11 as a whole and was not focused on how individual SES level may impact how a child defines a particular emotion.

Participants were recruited from three different schools: a private bilingual French–English school in New York, USA (School 1, *n* = 21), and two in Oxfordshire, UK (School 2, *n* = 14, and School 3, *n* = 14) (see Table 1 for participant school grade and gender per school). Schools were first contacted via email by the researcher, and for those that agreed to participate, parent/guardian consent forms and demographic questionnaires were sent to and shared by school staff with all families of students enrolled in grades 3 to 5. School staff returned completed consent forms and questionnaires to the researcher; a list of student participants sorted by classroom was then generated.

### 2.2. Materials

An audio recording device (a Tascam DR-05 Version 2 Dictaphone Linear PCM Recorder, New York, NY, USA) and a deck of 27 unruled A6 index cards (4″ × 6″) of varying colors were used to collect emotion definitions. Each card had one emotion word written in capital letters in black marker in the center, and there were no duplicate cards. The 27 emotion terms were the same as those used in Nook et al.’s (2017) developmental study of emotion concept representation: amazed, angry, annoyed, bored, calm, disappointed, disgusted, embarrassed, excited, grumpy, happy, hate, jealous, lonely, love, nervous, pleased, proud, relaxed, sad, safe, scared, sorry, surprised, thankful, upset, and worried.

At the time of data collection, Nook et al. (2017) was the only methodological precedent to the researchers’ knowledge relevant to this study’s design and its focus on child emotion definitions and the development of emotion concept representation. These emotion terms were deemed suitable for use as they varied in emotional valence (i.e., positive and negative emotions) and intensity (i.e., high and low), are considered to be understood in middle childhood in English (as based on the Baron-Cohen et al. (2010) study of emotion vocabulary acquisition ages), and could be defined within a 30 to 40 min session so as to not tax young participants unnecessarily.

### 2.3. Procedure

The following describes the general procedure adopted as data were collected at schools with different physical layouts. The researcher first collected participants from their classrooms to take them to a quiet ‘research’ room designated by school staff. The researcher and participant sat across from each other with a table between them, upon which the emotion card deck was placed. After an initial introduction, the researcher (a) explained the purpose of the study and that the child’s parent/guardian had given consent for them to participate; (b) confirmed information provided in the demographic questionnaire; and (c) obtained verbal assent, noting the child could stop the task at any time without penalty.

The researcher began the definitions task by asking the child to shuffle the emotion index cards into a deck, then passed back to the researcher. The researcher then explained they would present each card one by one, after which the child should say what they thought the emotion listed meant and that their response would be audio-recorded. The researcher also explained there were no wrong answers, the child could say as little or as much as they wanted, that they could say ‘Pass’ if they did not know the meaning of an emotion or did not want to provide a definition, and that only the researcher would listen to the recording.

Children were encouraged to ‘say the first thing that popped into their heads’ so that emotion definitions could be as spontaneous as possible and unbiased by the researcher, including semantic information that was most readily and easily accessed by child participants. This was in an effort to preserve a level of authenticity within a structured task without prescribing a particular strategy for children when defining emotions. The goal was for children to not feel limited in what they could say to describe emotions so that the study could report on verbatim accounts. An account of how children ‘naturally’ understand emotions through their language is relevant to how SEL programs structure discussions on emotions. The task was conducted in English by the researcher, but children were informed they could speak in French or Spanish throughout if they felt more comfortable, as these were languages understood by the primary researcher.

Once the child participant confirmed they understood and agreed to start the task, the researcher turned on the audio recorder, encouraged the child to speak into the recorder to maximize sound quality, and presented the first emotion card by reading out loud the emotion word listed. The researcher held up the emotion card in the child’s line of sight until the child had finished giving their definition and then placed the card face down on the table, presenting the next emotion card in the deck and reading out the emotion term listed. The procedure continued until all 27 emotion cards were presented. No further prompting occurred on the part of the researcher once the task began. If there were emotion words the child did not know, the researcher explained their meaning at the end of the task.

### 2.4. Analytic Procedure

#### 2.4.1. Data Preparation

Audio recording files of emotion definitions for each participant were transferred to the researcher’s laptop and then transcribed verbatim using the online software ‘Transcribe by Wreally.’ Once transcription was complete, files were deleted from the audio recorder. Transcription files for each participant were then downloaded as Microsoft Word documents, labeled with participant identification (ID) numbers, and removed from the ‘Transcribe by Wreally’ website. Transcripts were entered into an Excel spreadsheet using participant ID numbers and organized by target emotion term. The number of definitions in the corpus was 1239 (see Table S1 in Supplementary Material for number of definitions per age band). In instances where children produced an emotion definition in French or Spanish, the researcher translated these definitions into English prior to the analysis.

#### 2.4.2. Thematic Analysis of Child Emotion Definitions

To determine common themes mentioned across collected definitions, inductive thematic analysis was undertaken in line with the six-phase guide put forth by Braun and Clarke (2006) for rigorous and systematic thematic analysis. The six phases include the following: 1. Familiarizing oneself with the data; 2. Generating initial codes; 3. Searching for themes; 4. Reviewing themes; 5. Defining and naming themes; 6. Producing a report as a summary. The thematic analysis process for this study is presented below following these six steps. Themes were identified at the semantic level, meaning the researcher was concerned with the surface-level meaning of the content. This allowed for a descriptive organization of semantic content that was then followed by a level of interpretation of data to determine implications of which themes were frequent and not (Patton, 2014). All definitions were analyzed as a single corpus regardless of participant school, and as such, findings do not reflect independent descriptions of specific linguistic, educational and/or cultural contexts. Themes were identified based on frequent codes across the child emotion definition corpus and assessed for accuracy by a second reviewer (described in further detail below).

Definition transcripts for each participant were uploaded to NVivo 12 and read by the primary researcher as part of an initial round of exploratory coding (Phase 1) to note all mentions of specific scenarios, topics, and/or individuals (Phase 2). These codes were grouped into categories where possible as part of an iterative process (Phase 3). A codebook was then generated by and downloaded from NVivo as a Word Document comprising an alphabetical list of all codes organized into their respective categories; the ‘spread’ of a given code indicating the number of files in which it appeared (reflecting how many child participants made mention of a particular code in their definitions); and the number of overall references to a particular code (which reflected how many times one child participant referenced the codes across their emotion definitions).

Codes and code categories appearing in transcripts produced by close to half of child participants (*n* = 24) were flagged as common and used to generate nine initial themes and sub-themes (Phase 3). As half of the number of participants was 24.5, it was decided by researchers not to round up to 25 participants as a threshold for how many child participants needed to reference a code for it to be considered common in the definition corpus. This threshold was adopted as an operational rule to identify the most frequent patterns across definitions and is not a measure of qualitative relevance. Codes mentioned by close to half of child participants were used as the basis for themes to determine the most frequent content in the definition corpus—the focus of this exploratory study’s first research question. One emotion definition could be coded to multiple themes in the emotion framework based on different portions of text within a single definition. Coding to themes was treated with equal weight across themes (see Table 2 for definition examples, coding decisions, and analytic explanation).

Themes and sub-themes were then reviewed by the primary researcher and a second reviewer by comparing them to coded portions of emotion definition text to assess fit (Phase 4). The second reviewer received a portion of all child definitions (25%, totaling 310 definitions) that was randomly selected and had already been coded by the primary researcher, as well as theme descriptions to complete the external review of fit for generated themes. The second reviewer assessed the conceptual fit of the primary researcher’s coding decisions to the framework to determine if they were accurate by performing the following: 1. looking at the portion of coded text in the emotion definition (highlighted) used by the primary researcher to make a decision and the theme assigned; 2. reading the theme description; and 3. determining whether coded content fit the theme description. Coded content was determined to fit with a theme if it was conceptually covered by the theme description. The second reviewer was also asked whether they had thought of additional themes when reading through definitions and if the language for theme descriptions was clear. Sub-themes generated by the primary researcher for the same portion of definitions were also assessed by the second reviewer via the same process.

The second reviewer did not propose additional themes or sub-themes as they deemed themes and sub-themes identified by the primary researcher to be sufficient in capturing topics mentioned in child emotion definitions. The second reviewer also deemed the primary researcher’s coding decisions for the initial nine themes to be accurate (in that the second reviewer noted they would have made the same coding decisions had they coded the definitions independently). As such, there were no disagreements with the primary researcher’s coding decisions.

Instead, discussion between the primary researcher and second reviewer centered on the clarity of theme names and descriptions, namely, how to make them more informative. This resulted in two themes being combined and a final description of eight themes, with accompanying sub-themes, organized hierarchically (Phase 5; see Table S2 for sub-themes). The final eight themes were summarized with examples from collected child definitions (Phase 6; see Table 3). Regarding the two themes that were combined, the initial themes ‘occurrence of non-emotional human behavior’ and ‘physical experience’ were combined based on conceptual overlap into one theme, ‘observable signs,’ to capture references to an observable sign that could be physical or sensory, tied to human behavior. Child emotion definitions originally coded to the themes ‘occurrence of non-emotional human behavior’ and ‘physical experience’ were recoded as references to the theme ‘observable signs.’

#### 2.4.3. SEL Program Mapping to Child-Informed Emotion Theme Framework

The eight themes identified through the study’s thematic analysis constituted the child-informed emotion framework used to map SEL program content. The SEL programs (a total of 38) were those used in studies identified in a previous systematic review by O’Grady and Nag (2024) of emotion-focused content in school-based SEL programs universally implemented in Grades 3 to 5 (a total of 54 studies that used 38 SEL programs, meaning some studies included in the review used the same SEL program).

SEL program lesson topic names, aims, and activities were listed by program in an Excel spreadsheet and then coded to framework themes reflected as labels of header columns, as this was the level of information consistently available across studies. SEL program content was extracted from what was reported in published SEL program studies included in the O’Grady and Nag (2024) systematic review. To complement information reported in studies (whether in main text or as Supplementary Material), an online search of SEL programs was conducted to see if handbooks were available. Handbooks for SEL programs included in this study were behind paywalls, and as such, researchers had to rely on what was reported in SEL program studies themselves (which included all lesson names/topics and the general goal of an SEL program).

A coding value of 1 in the mapping spreadsheet indicated an SEL program component (e.g., lesson topic) targeted a framework theme. One program element could be coded to multiple themes (a summary mapping table that lists all programs and their respective components reported in studies as well as code descriptions can be accessed on the Open Science Framework (https://osf.io/azh9x/files/5myjf?view_only=51895993f5334a38a2313af0709ee910). Mapping was primarily based on the focus of a lesson or unit’s topic, using additional information from lesson activities when reported in studies, but this was rarely included in studies. Overall, the following information was reported for SEL programs (see Table 4):

The content and level of detail available in descriptions for SEL program components varied among SEL programs, and so it was decided the analysis would be constrained to indicate whether an aspect of an SEL program addressed an emotion theme or not. An SEL program was counted as targeting a theme in the framework if at least one program component (e.g., lesson name, lesson focus) was mapped to it. Across programs, lesson/unit names and/or descriptions of their focus provided a sufficient level of detail to map to themes in the child-informed emotion framework using theme definitions. Even if multiple components targeted the same framework theme, the SEL program was still only counted once for targeting a particular theme. Given incomplete information on SEL program content, mapping did not indicate the extent to which a particular emotion theme was addressed within one SEL program, whether the number of lessons in which it appears, the depth with which it is developed, the time devoted to it, or its importance within the program.

To complete this study’s mapping exercise, the primary researcher and a second researcher trained in child social–emotional development and interventions independently mapped all SEL programs to mitigate potential biases in the interpretation of reported SEL topics addressed by lessons. To map an SEL program component, coders consulted the description of each theme and assessed if the focus of the component matched. Discussion allowed coders to share instances of uncertainty and a rationale for coding decisions so that there was unanimous interpretation for all SEL program content reviewed. Discussion also ensured coders did not over-extrapolate or make assumptions about the core focus of an SEL program component. Coding disagreements were also discussed to reach final mapping decisions via consensus before mapping trends were analyzed and summarized in frequency tables. Table 5 provides examples of the decision process for mapping components in three different SEL programs.

## 3. Results

### 3.1. Common Themes Across Child Emotion Definitions

Thematic analysis revealed children in the study sample referenced the following themes in their emotion definitions: ‘interpersonal dynamics’ (100% of participants), ‘non-social situations’ (84%), ‘observable signs’ (82%), ‘performing an activity’ (69%), ‘describing a location’ (67%), ‘being a student’ (59%), ‘partaking in an organized event’ (53%), and ‘cognitive states’ (53%) (see Table 2 and Table 3).

### 3.2. Mapping of SEL Programs to Child Emotion Theme Framework

Lesson/unit names, aims, and activities in SEL programs, depending on what was reported, were the focus for the mapping exercise to address this study’s second research question. Mapping to the child-informed emotion framework indicated SEL programs frequently addressed the following: ‘interpersonal dynamics’ (97%), ‘cognitive states’ (95%), ‘performing an activity’ (87%), and ‘observable signs’ (71%). Themes targeted by 34% or less of SEL programs were ‘partaking in an organized event,’ ‘being a student,’ ‘non-social situations,’ and ‘describing a location’ (see Table S3). On average, a single SEL program targeted five out of the eight themes in the framework and at minimum two (a spreadsheet featuring raw frequency counts of SEL program mapping is available on the Open Science Framework at this link (https://osf.io/azh9x/overview?view_only=51895993f5334a38a2313af0709ee910).

## 4. Discussion

This study analyzed child emotion definitions using thematic analysis to develop an initial framework to describe how a group of children in a specific age band think about emotions, to which emotion-focused content in SEL programs for the same age band was then mapped. The aim was to attempt to bridge how emotions are taught in the classroom throughout middle childhood with how a sample of children experience them (as indicated by child-produced emotion definitions), allowing for representations of emotion shift across different phases of child development. The study is the first, to the researchers’ knowledge, to build a corpus of child-produced emotion definitions specific to middle childhood. Two research questions were addressed: Are there common topics that children ages 8 to 11 reference when defining emotions? Does SEL program content on emotions for the same age band address these topics?

The study was exploratory, and definitions were produced by a small sample of children ages 8 to 11 that cannot be said to reflect the general population of children in this age band. However, the data collected and study findings constitute the beginning of building an evidence base for child-produced emotion definitions to glean how children think about and understand emotions in their own words, as reported in a non-clinical setting. Furthermore, these preliminary findings regarding which topics commonly feature in a sample of child emotion definitions are useful for an initial dialogue with emotion-focused content in SEL programs that are implemented in middle childhood, but do not constitute a call for specific changes to the design of SEL programs reviewed.

### 4.1. Common Themes in the Study’s Child Emotion Definition Corpus

In their emotion definitions, almost all child participants (at least 82%) referred to interpersonal dynamics, experiencing a non-social situation, and observable signs that indicated non-emotional human behavior. Children in this study often relied on specific experiences (e.g., cultural events), environments, and social situations to make meaning of emotions in their definitions. This lends support to emotion theorists who stress the importance of interactions and culture in shaping emotion concepts and emotional experience, such as Averill (1980) and Harré (1986). Still, the study sample does not reflect the general population of children in middle childhood, and so we do not extend findings of recurrent themes here to apply to emotion definitions produced by children ages 8 to 11 in general. Topics raised in definitions here are necessarily drawn from the experiences of children in the sample, which occur in specific environments and cultural contexts.

Furthermore, the language used to discuss emotions and having access to words across different languages to describe one emotion are important factors that would impact how children define emotions. Children in this study came from monolingual and multilingual backgrounds, and as mentioned earlier, some switched languages when defining certain emotions. However, thematic analysis was conducted at the semantic level of emotion definitions; all definitions were translated to English prior to analysis, and analysis was not focused on specific word usage.

Analyzing cross-cultural variation or how language background impacted the content of emotion definitions was beyond the scope of this study, as it was concerned with what was shared, from a conceptual content perspective, across its sample of children ages 8 to 11, but this would be an interesting avenue for future research with larger samples. Only participant ID numbers were linked to emotion definitions to organize them, with no additional demographic information included. Analysis of the emotion definition corpus was focused on setting precedent for identifying thematic commonalities in child emotion definitions. Although the study cannot speak to how the linguistic profile of children influenced the way they defined emotions, there was evidence of the potential influence of context and culture to anchor how children in this sample understand emotional experience. Just over half of children (53%) named specific organized events to define different emotions, including mentions of holidays, the practice of celebrating birthdays, and events organized by their school community (e.g., a sports/field day).

Within the theme of ‘interpersonal dynamics,’ nearly all participants mentioned the sub-theme of a specific social situation that involved positive behavior from others (e.g., receiving a gift, help, or a compliment). The next common sub-theme/topic within ‘interpersonal dynamics’ was the experience of being alone. The fact that ‘interpersonal dynamics’ was a theme found in transcripts produced by all child participants aligns with the stance that emotions constitute a form of social information, as put forward by Van Kleef (2016). In this perspective, the focus is on how emotions are experienced in the presence of others, and how the emotions of others can impact an individual’s feelings, thoughts, and actions in turn. Such an interpersonal perspective on emotion is also summarized in the ‘Emotions as Social Information’ Model (Van Kleef, 2009).

Regarding the theme of ‘non-social situations’ that captured reference to specific events or scenarios, child participants most often referred to disappointing or negative events in their emotion definitions and alluded to some aspect of danger or safety. The recurrent mention of negative events by children in this study across their definitions, even when emotions being defined were positive, aligns with research findings that negative events are considered more emotionally arousing or particularly salient (García-García et al., 2016). Child emotion definitions in this study were spontaneously provided, so it is plausible that definition content reflects information most salient and readily accessible to participants for a given emotion.

Recalling personal events also tends to generate more mentions of negative emotions and of mixed blends (i.e., emotions of opposing valence) (Salas et al., 2012). Although children in this study were not asked to share personal experiences to define emotions, it is not unreasonable to assume children drew upon their lived experiences, that is to say, ‘personal events,’ to describe emotional states. Furthermore, negative experiences may have come to mind quicker for child participants, as negative experiences have been found to be more readily encoded into memory and easier to retrieve due to their association with stress and high arousal (Hoscheidt et al., 2014; Kensinger, 2009).

Being injured or physically unwell can also be considered an emotionally heightened situation and, as such, it is unsurprising that it was spontaneously mentioned in child emotion definitions sampled here. Within the theme of ‘observable signs,’ approximately 78% of children in this study mentioned a physical or sensory experience. Twenty child participants (approximately 41% of the sample) referred to physical injury when defining angry, annoyed, sad, safe, sorry, thankful, upset, and worried. The link between negative physical experiences (being physically hurt) and emotional states is similar to children’s frequent mention of disappointing or negative events discussed earlier.

Another interesting sub-topic for physical/sensory experience was sleep, mentioned by 21 child participants (close to 45% of the sample). Sleep can be considered a state where a child no longer consciously engages with others or tasks, but in which emotional experience can still arise, as evidenced by research on the experience of dreams relayed through child dream reports (Sándor et al., 2016) and nightmares (Scarpelli et al., 2019a; Secrist et al., 2019), for example. Sleep has also been linked to emotion regulation (Carcelén-Fraile et al., 2026; Marano & Mazza, 2026) and emotion processing in children (Kurdziel et al., 2018) as well as adults (Scarpelli et al., 2019b). Children here did not discuss dreams and nightmares in their emotion definitions but brought up sleep when defining a variety of emotions including bored, calm, embarrassed, grumpy, love, relaxed, safe, and upset. Children in this sample referenced sleep when conveying a calm, peaceful internal state and describing contexts tied to sleeping, both positive and negative: being bored when others are asleep and they cannot engage; being embarrassed when they had a secret that they slept with teddy bears which was then found out; being excited at the thought of a sleepover at a friend’s house; being grumpy upon waking.

Taken together, study findings for the first research question emphasize child participants’ spontaneous considerations when providing emotion definitions of whether an emotion is positive or negative; and whether the emotion is linked to an intense or subdued sensory experience. This links back to previous conceptual models of emotion that construe emotions along two dimensions—one of valence, with emotions ranging from positive to negative, and one of arousal or intensity (see the Plutchik wheels of emotion (Plutchik, 1958, 1960, 1991); the Russell (1980) Circumplex Model of Affect; the Bradley and Lang (1994) Valence–Arousal–Dominance Model of Emotion; and the Cambria et al. (2012) Hourglass Model of Emotion).

### 4.2. Mapping SEL Programs to a Child-Informed Emotion Theme Framework

With regard to the second research question, all topics in the child-informed emotion framework were addressed by at least one component (e.g., lesson, unit) of SEL programs reviewed here, based on content description provided in intervention studies. On average, a single SEL program addressed five out of the eight themes in the framework and at minimum two.

As information on SEL program content was brief and inconsistently reported across programs, findings serve only to launch a theoretical comparison that describes which topics SEL programs address in the child-informed emotion framework. Differences between the frequency of certain themes across SEL programs and the study’s child emotion definition corpus are not taken as an indication of how SEL program content should be adapted, as this goes beyond the current analysis and cannot be supported by the data. It must also be acknowledged that the child emotion perspective presented here through the framework is limited to that of the group of children who participated in the study and could easily shift if different children completed the emotion definition task.

The way emotions are discussed in the classroom through SEL programs also differs from how emotions are apprehended in the study’s definition task. Child participants in the task were focused on conveying their interpretation of what an emotion means—a purely reflective and communicative exercise. SEL programs reviewed here had the goal of promoting specific skills in children in a classroom setting, namely, to identify one’s own emotions and to regulate them to avoid negative social interactions with peers and adults, and to not disrupt learning in the classroom. Furthermore, SEL programs will combine content familiar to children with novel information as a pedagogical strategy to support learning. In contrast, children’s understanding of emotion as expressed through emotion definitions reflects conceptual information already acquired.

Nearly all emotion-focused content described in SEL programs addressed interpersonal dynamics. This is not surprising given SEL programs are rooted in acknowledging the interaction between and intertwined development of social and emotional skills. Non-emotional ‘cognitive states’ were also a frequent topic included in 95% of programs. This aligns with the primary aim of SEL programs to teach skills to children that promote positive coping and overall social–emotional development, and relates back to regulatory strategies for internal states (not necessarily emotional or affective). ‘Performing an activity’ was another topic commonly addressed in mapped SEL programs (87%). This mapping finding is unsurprising as SEL program content often relies on scenarios to introduce discussion on emotions.

A majority of SEL programs (71%) also focused on observable signs of human behavior, primarily physical, when describing emotions. Many SEL programs reviewed here incorporated physical and/or external cues to build an understanding of emotional states for children ages 8 to 11. SEL programs included lessons focused on noticing external ‘body’ clues and body language, changes in breathing, and other physiological manifestations of emotions (referenced either in the self or others) to support children to better understand emotional experiences.

Facial expressions in particular were frequently mentioned in SEL program content descriptions. Reflecting briefly from a pedagogic perspective, facial expressions can serve as a useful starting point to anchor discussion around identifying emotional states, as they can be perceived first. Facial expressions are an externalized display of emotion, a context clue that can be used by children to identify emotional states (e.g., Cuciniello et al., 2025; Romani-Sponchiado et al., 2022). Using facial expressions in SEL program lessons reinforces information that children in this age band are expected to have acquired (Pons et al., 2004) before introducing either more nuanced reflection or more complex scenarios that can give rise to a particular emotion.

The importance of context to the development of child emotion understanding is underscored by evidence of cross-cultural differences in emotion socialization practices and displays (Carmiol & Schröder, 2026; Chan et al., 2026; Cho et al., 2022; Friedlmeier et al., 2011; Möller et al., 2022; Zhou et al., 2023). Emotion socialization refers to how rules and norms about the way emotions are expressed and communicated are shared in a community, both implicitly and explicitly. Zahn-Waxler (2010) defines emotion socialization as the environmental influence that informs how emotions are expressed, regulated, experienced, and understood from childhood to adolescence. This has important implications for the choice of scenarios used in SEL programs to illustrate emotional events. Relatability to program content can promote and maintain engagement with SEL programs, including that of students and practitioners, in the same way it has been discussed in culturally responsive teaching research (e.g., Gay, 2015; Ladson-Billings, 1995).

Here, reported SEL program lesson descriptions that were available referenced situations in general terms (e.g., ‘imagining the situation presented by the teacher,’ ‘the application of mindfulness to everyday situations’). Access to the specific scenarios used in lessons for all SEL programs was not possible, and so the study cannot speak to whether culturally specific events or culturally responsive teaching was used to teach children about emotions. However, when situations were referenced with greater specificity in SEL program descriptions, these situations were primarily negative or stressful. Most notably, interventions to teach mindfulness had clear lesson topics that conveyed the valence of a situation without adding more specifics (e.g., ‘cope with situations/events that provoke anxiety,’ ‘to make visible the unarticulated thought process that occurs in someone’s mind in an annoying social or emotional situation,’ ‘recognizing a social problem situation’). This suggests SEL programs reviewed here had a focus on supporting children in regulating their own negative emotions and coping with those of others.

### 4.3. Limitations and Future Directions

Although the study was exploratory, it is important to consider that emotion definitions were produced by a small sample of children that, although diverse, cannot be said to reflect the general population of children ages 8 to 11. It would be of interest for future research to test whether the common topics found here across emotion definitions are maintained with a larger sample of children in middle childhood. This would allow us to better contextualize and interpret this study’s preliminary findings.

Concerning the analysis of child emotion definitions, thematic analysis is also a method that hinges on researcher interpretation of language, which unavoidably comes with a degree of subjectivity. The study was concerned with identifying frequency patterns of topics found in emotion definitions produced by a sample of children. Thematic analysis served to achieve this goal by providing initial descriptive data and plausible themes grouping topics mentioned in brief definitions. Different researchers may have produced different topic codes and themes, and as acknowledged earlier, different children may have frequently referenced other topics in their emotion definitions due to their individual experiences. Providing causal explanations for why the sample produced emotion definitions the way it did is beyond the scope of this study. The present study’s child-informed framework of emotion themes is solely understood as a preliminary frame to bridge reflection on emotion-focused SEL program content and to complete a comparative exercise.

Another important limitation is that definitions were analyzed in a single language, English. Although some children spontaneously provided definitions in different languages depending on the emotion being defined, these non-English definitions were translated to English prior to analysis. As such, findings can only be said to pertain to themes found in English for the study’s sample. Similar analysis of emotion definitions produced by children who speak languages from different language groups would be of interest for future research on cross-cultural comparisons to identify themes shared across child emotion definitions in general. This would allow for a more holistic understanding of how children talk and experience emotions across different phases of childhood and in different contexts, which can then inform scenarios used in emotion-focused SEL program content in turn.

Although this study supports the importance of accounting for children’s individual lived experiences in shaping their emotions, it acknowledges the oversight of not incorporating demographic factors (namely SES) within its thematic analysis. It would be of interest for future studies on child emotion definitions to collect data on SES from diverse groups, and with larger sample sizes, to better speak to linguistic trends across child populations. This would allow for a richer analysis of topic trends in emotion definitions across different groups of children to determine which themes are potentially maintained in how children talk about emotions. It would also contribute sufficient data to consider potential differences between multilingual versus monolingual speakers in how they define emotions.

Regarding the study’s SEL program mapping, the lack of access to complete SEL program manuals and uneven level of reporting of program components were important limitations to the analysis. Study findings are based on brief descriptions of SEL program content that was limited to what was included in intervention studies, which was also inconsistent regarding level of detail (e.g., some studies reported all lesson names and their focus or aim while others only described topics covered by the program in general terms). Programs reviewed here also do not reflect all SEL programs available to teach emotions in middle childhood. As such, findings from the program mapping are only a starting point to describe how content dedicated to teaching children ages 8 to 11 about emotions addresses topics mentioned in emotion definitions produced by children in the same age band. The mapping exercise cannot be considered comprehensive. Future research on SEL program content needs to be able to access manuals to have a more in-depth and complete understanding of the design of emotion-focused content in SEL programs, especially noting the activities chosen for specific lessons.

It would also be of interest to identify the level of emphasis for topics both within and across SEL programs when teaching emotions, something the present study was unable to do in its current analysis. Exploring the depth at which SEL programs cover an area of child emotion understanding through different program components would align with research work dedicated to identifying core components of SEL programs (Frye et al., 2024; Lawson et al., 2019; Wigelsworth et al., 2023, 2024; see Shi & Cheung, 2024 for meta-analysis of effective SEL program components in general). Such evidence, based on more detailed content analysis, could better support practitioners in choosing programs for their context as well as implementation research in explaining SEL program impact. Meaningful work has also been performed in this area by the EASEL lab at Harvard University, USA, to identify to what degree different social–emotional skills are targeted across SEL frameworks (see the ‘Explore SEL’ tool http://exploresel.gse.harvard.edu/, accessed on 6 August 2026).

## 5. Conclusions

The study presents descriptive information as part of an initial exploration of how a sample of children in middle childhood defines emotions, and of the topics addressed by emotion-focused content in SEL programs for the same age band. Study findings indicate context and a social dimension were recurrent topics in how children ages 8 to 11 in this sample defined emotions. Almost all SEL programs reviewed referenced interpersonal dynamics, echoing the social dimension to emotion found in the study’s child emotion definition corpus. Study findings prompt reflection on which scenarios are selected in SEL programs to illustrate emotional states but cannot speak to their design. By providing an initial account of child emotion definitions and linking them to SEL programs, this exploratory study contributes preliminary insight into how some children ages 8 to 11 think about emotions and offers a method for using a child-informed lens when considering how emotions are taught in middle childhood through SEL programs. This is part of a broader discussion of SEL program content being able to reflect the experiences of the communities in which they are implemented that can be taken up by future research.

## Figures and Tables

**Table 1:** Participants by school, school grade, and gender.

	School 1		School 2		School 3	
	Male	Female	Male	Female	Male	Female
Grade 3	5	3	3	2	7	7
Grade 4	4	1	3	6	0	0
Grade 5	4	4	0	0	0	0
Total	13	8	6	8	7	7

**Table 2:** Examples of collected child emotion definitions transcribed verbatim, randomly selected, with analytic explanation of coding decisions.

Child Emotion Definition with Underlined Text Used in Coding	Coding Decision and Explanation
Surprised: You’re surprised with presents. So that you’ve just got a good feeling that. So many presents you had.	Definition of surprised coded to theme ‘non-social situations’ based on underlined text (“with presents” and “So many presents you had.”) that references a scenario of receiving a gift. Further context is not specified, including the person who may have given the present or the relationship between the gift-giver and gift-receiver, and as such, was not coded to the theme ‘interpersonal dynamics’ in addition. The text ‘Got a good feeling’ is also a main part of this definition; however, a feeling is an internal state considered affective/emotional and, as such, would not qualify as being coded to the theme ‘cognitive states.’ In essence, the child has referenced an emotional state to define an emotion, which is a topic, but not an informative one. The goal of the study’s primary research question was to uncover common topics across child emotion definitions. It was decided between the authors that reference to an emotional/affective state is inherent to the act of defining an emotion, meaning it would apply to all child definitions thematically and as such, would not offer additional insight useful to the study’s analysis.
Excited: When you’re quite like like let’s say you’re going to the Harry Potter studios, you’d be really happy and it like you just won’t stop moving.	This definition of excited was coded to the themes ‘non-social situations’ and ‘describing a location’ based on the underlined text “you’re going to the Harry Potter studios” as it references a specific scenario and precise location; as well as to the theme “observable signs” based on the text “you just won’t stop moving” as this references a physical behavior (human) that is observable. Again, the text “you’d be really happy” is another part of this definition; however, it references another emotion/affective state which, as explained in the above example, was expected to be mentioned across child emotion definitions given the nature of the task and not included as part of the child emotion framework as a separate topic.
Relaxed: When you have nothing to do, I can just take a good book and you’re relaxed.	This definition of relaxed was coded to the theme ‘performing an activity’ based on the underlined text “take a good book” as it references an individual doing something and a specific activity; and to the theme ‘non-social situations’ based on the text “when you have nothing to do” that references a situation describing a lack of activity, but a scenario nonetheless that does not involve other individuals or social interaction (and is also why it was not coded to the theme ‘interpersonal dynamics’).
Hate: When you don’t like something you… really don’t like it it’s… it’s you can’t do it it’s… it’s too bad uh… maybe sometimes something bad happened and so you don’t want to do it anymore.	This definition of hate was not coded to any themes in the framework. Text fragments “don’t like something” and “don’t want to do it anymore” refer to liking something/having a preference and want, in the sense of desire, that are both considered affective states. The text “can’t do it” implies doing something, but a specific activity is not named and so, the text was not coded to “performing an activity.” The text “Something bad happened” is a reference to a situation but is not specific enough with more details of a situation or context to be coded to ‘non-social situations.’

**Table 3:** Common themes in collected child emotion definitions transcribed verbatim, with theme description and examples of child emotion definitions.

Theme	Description	Example (Verbatim with Coded Portions Underlined)
Interpersonal dynamics	Definition references relationships, communication, or behaviors relating to others, or a social situation.	“[Embarrassed is when] you feel like… like that everybody is on like someone’s side and like they are like against you. And there’s humiliating things. I once remember someone someone’s was trying um to do it to me, but it didn’t work. So so um they were trying um to humiliate me, but I guess um um I’m um humiliating him. So it didn’t work.”
Non-social situations	Definition references a specific scenario or context that is not social in nature.	“[…] There’s nervous like when you get like… maybe hit and run, and then you’re waiting for the police to like come ‘cuz like maybe they broke your tire or something, I don’t know, and you’re just scared, and so you have all the butterflies in your stomach… and you don’t know what’s gonna happen next, but it’s like more of a serious matter than a test uh test and… that’s nervous, I guess.”
Observable signs	Definition references an observable sign (physical or sensory), tied to human behavior.	“[Grumpy] it’s for example sometimes when you wake up when your mom needs to wake you up in the morning, you’re very grumpy because you sometimes you talk with your a little bit with your mouth closed. So… you know. You moan… and so you don’t you don’t feel very happy.”
Performing an activity	Definition references a specific activity that may or may not result in experiencing the emotion being defined.	“[Disappointed is] when… you were sure something was going to happen… but well… basically when you thought something was going to happen, like something interesting, for example if you watch a soccer game, you want a team to win, but maybe it will lose, so you would say, ‘Oh la la I was sure they were going to win.”
Describing a location	Definition references a specific location where one might experience the emotion being defined.	“[Relaxed is] when you… like if you’re at the beach and… and… and… lll like in the sun relaxing.”
Being a student	Definition references the experience of being a student or a topic that is tied to schools or an academic context.	“Nervous is when… it’s sort of like when you have a test, and you’re like really nervous ‘cuz you studied all night, but you’re like, ‘Oh yeah, I know everything.’ but you’re just nervous because you get butterflies in your stomach, […] like you make up all these crazy scenarios in your head […]”
Partaking in an organized event	Definition references a specific event; includes holidays and celebrations.	“[Proud is] When you’ve done something good or you’ve won something. I was proud when I won a medal on Sports Day which was last which was on a Monday. And I was first.”
Cognitive states	Definition references an internal state, not emotional or affective; relates to self-regulation, perspective-taking, or thinking about the emotion being defined.	“[Angry is when] you can’t control your emotions about something.”

**Table 4:** SEL program content reported in studies for *n* programs (out of 38).

Description of lesson foci + activities for program as a whole	Description of lesson foci + partial description of activities for lesson foci (≥40%)	Lesson names; description of lesson focus; no activities	Unit names; description of unit focus + activities per unit	Description of lesson foci; no activities
8	4	3	3	2
Unit names; description of lesson foci; partial description of activities per unit (≥50%)	Description of lesson foci + activities for 1 lesson focus	Description of lesson foci + activities per lesson focus	Description of unit and lesson foci + activities for program as a whole	Description of unit and lesson foci; no activities
2	1	1	1	1
Lesson names; description of activities per lesson	Lesson names; description of lesson focus + activities per lesson	Lesson names; no activities	Unit and lesson names; description of activities for 1 lesson	Unit and lesson names; description of lesson foci; no activities
1	1	1	1	1
Unit and lesson names; description of unit and lesson foci + activities per unit	Unit and lesson names; description of activities for 60% of lessons	Unit names + activities per unit	Unit names; description of unit focus; no activities	Unit names; description of lesson foci + activities for program as a whole
1	1	1	1	1
Unit names; partial description of lesson foci + activities per unit (67%)	Unit names; no activities			
1	1			

**Table 5:** Examples of the decision-making process for mapping program components of three SEL programs.

Program Name/Sample Component (Extracted from Study Article)	Interpretation	Decision
Aussie Optimism Program: Feelings and Friends Lesson: Body clues and first aid for feelings Aim: For students to appreciate physiological manifestations of various emotion; learn emotion regulation skills; be introduced to the idea that there are strategies that can be used to cope with unpleasant feelings and learn to self sooth.	Lesson considers the link to physiological aspect to emotions and presents the idea of regulation of emotions, meaning thinking about emotions and strategies that are not necessarily affective to regulate emotions and are more cognitive; this focus matches the themes that reference a physical or sensory sign, as well as self-regulation; although performing an activity could be a strategy to regulate emotions, an activity is not explicitly stated in the description of the lesson.	Code lesson to two themes: ‘observable signs’ that captures reference to a physical or sensory sign that is tied to human behavior; and ‘cognitive states’ that covers reference to an internal state not emotional or affective, self-regulation, perspective-taking, and thinking about an emotion. Do not code to ‘performing an activity.’
Bullying Literature Project—Moral Disengagement Lesson aim: Encourage awareness about bullying.	Primary focus of lesson, based on its title, is on the situation of bullying and raising awareness about bullying, which means what bullying may look like/how it can happen and between who. Bullying is a reference to relationships between people. No mention of specific scenario or context that is not social in nature.	Code lesson to ‘interpersonal dynamics’ that covers all reference to relationships, communication and behavior relating to others, and social situations. Do not code to the theme ‘non-social situations’ that is reserved for references to a specific scenario or context that is not social in nature.
EDI Program: Would you like to travel around the planet of emotions? Unit: Intrapersonal skills Aim: To teach students how to know and understand their own and others’ emotions. Description: Covered topics such as perceiving emotions, the body and the mind, being aware of one’s own emotions, identifying and discriminating emotions, adequately expressing emotions, differentiating one’s own. emotional states from others’, and learning to accept, respect, and love one another.	Based on the unit’s name, aim, and descriptions, lessons in the unit touch on relating to others and the ability to identify and communicate about emotions, which would involve thinking about an emotion. The lesson topic description also references behaviors towards others such as respect and acceptance and perceiving the body and the mind, which link to physiological/physical states (potentially observable) in reference to the body as well as internal states that are cognitive and not necessarily affective in its reference to the mind.	Code unit as targeting the theme ‘interpersonal dynamics’ that covers mentions of relationships, communication and behaviors relating to others, and/or a social situation; the theme ‘observable signs’ and ‘cognitive states.’

## Data Availability

For access to the child emotion database described in this study and the SEL program mapping table, please visit the project page on OSF: https://osf.io/azh9x/overview?view_only=51895993f5334a38a2313af0709ee910.

## Supplementary Materials

The following supporting information can be downloaded at: https://www.mdpi.com/article/10.3390/bs16091482/s1, Table S1: Number of emotion definitions per participant age band; Table S2: Common themes and topics across child emotion definitions in the corpus; Table S3: Results of SEL program mapping and references in child emotion definitions to themes in child-informed emotion framework (in percentages).

## Abbreviations

The following abbreviations are used in this manuscript:

ID	Identification
SEL	Social-emotional learning
SES	Socio-economic status
