# Supplementary material for: Bridging Emotion in the Classroom with Child Understanding: Initial Findings from Linguistic Analysis for SEL Programs
Source: Behav Sci (Basel). 2026 Aug 25;16(9):1482. doi: 10.3390/bs16091482 (PMC13603433; doi:10.3390/bs16091482)
Supplement: Supplementary file 1 [file behavsci-16-01482-s001.zip › behavsci-4417829-supplementary.pdf]

Supplementary Materials

Table S1. Number of emotion definitions per participant age band.

Age band (years)	Definitions (n)	Omitted definitions (n)*	Total participants (n)	Participants who omitted definitions (n)
8 to 9	279	17	11	3
9 to 10	637	11	24	6
10 to 11	377	1	14	1
**Total**	**1,239**	**29**	**49**	**10**

*Refers to instances where participants did not provide a definition; emotion terms that were undefined by some participants included: disappointed, disgusted, jealous, pleased, proud, scared, surprised, and thankful.

Table S2. Common themes and topics across child emotion definitions in the corpus.

Theme	Transcripts (n)	Frequent sub-themes (n transcripts)	Sample topics (n transcripts)
Interpersonal dynamics	49	Mention of specific social situation (48)	Positive behavior from others (35)
			Being alone (23)
			Negative behavior from others (20)
			With family (15)
		Isolation, rejection (28)	No one to play with, being left out (19)
		Conflict (24)	Hurting someone's feelings (12)
Non-social situations	41	Disappointment, negative event (25)	Doing something wrong/bad (14)
		Danger, safety (22)	Being in danger (13)
Observable signs	40	Physical, sensory (38)	Sleep (21)
			Injury (20)
		Effort (18)	Doing nothing (14)
Performing an activity	34	Leisure (32)	Playing (19)
			Physical/sport activities (15)
			Engaging with media entertainment (e.g., watching TV/a film, reading a book) (14)
Describing a location	33	Outdoor environment (16)	The dark (5)
		Being at home (10)	Being in bed (5)
Being a student	29	Going to school (14)	Being at school (11)
		Having an assessment (9)	Receiving a school mark/grade (good/bad, failing) (9)
		Teacher (9)	Description of teacher behavior (e.g., assigning homework, interacting with parents) (9)
Partaking in an organized event	26	Birthday (16)	Having a birthday (party, surprise) (16)
		Performance (12)	Participating in a stage performance (12)
Cognitive states	26	Desire (18)	Wanting something (object), to do something (12)
		Nerves (15)	Having stage fright (9)
			Having 'butterflies in your stomach' (5)

Table S3. Results of SEL program mapping and references in child emotion definitions to themes in child-informed emotion framework (in percentages).

	**Being a student**	**Cognitive states**	**Describing a location**	**Interpersonal dynamics**	**Non-social situations**	**Observable signs**	**Partaking in an organized event**	**Performing an activity**
SEL programs (%)	32	**95**	26	**97**	32	**71**	34	**87**
Child participant mentions in emotion definitions (%)	59	53	67	**100**	**84**	**82**	53	69
